# A functional role of Ephrin type-B receptor 6 (EPHB6) in T-cell acute lymphoblastic leukemia

**DOI:** 10.1186/s40364-023-00531-3

**Published:** 2023-10-20

**Authors:** Mattia Colucci, Nadia Trivieri, Gandino Mencarelli, Elisabetta De Santis, Francesca Sansico, Francesco Tamiro, Alberto Visioli, Chiara Barile, Riccardo Pracella, Giovanni Rossi, Elena Binda, Vincenzo Giambra

**Affiliations:** 1 Hematopathology Unit, Institute for Stem Cell Biology, Regenerative Medicine and Innovative Therapeutics (ISBReMIT), Fondazione IRCCS “Casa Sollievo della Sofferenza”, Viale Padre Pio, 7, 71013 San Giovanni Rotondo (FG), Italy; 2 Cancer Stem Cells Unit, Institute for Stem Cell Biology, Regenerative Medicine and Innovative Therapeutics (ISBReMIT), Fondazione IRCCS “Casa Sollievo della Sofferenza”, Viale Padre Pio, 7, 71013 San Giovanni Rotondo (FG), Italy; 3 StemGen SpA, Milan, Italy; 4 Department of Hematology and Stem Cell Transplant Unit, Fondazione IRCCS Casa Sollievo della Sofferenza, Viale Cappuccini, 1, 71013 San Giovanni Rotondo (FG), Italy

**Keywords:** T-ALL, EphB6, Single-cell RNA-Sequencing, Leukemia-initiating cells (LICs), Biomarkers

## Abstract

T-cell lymphoblastic acute leukemia (T-ALL) is an aggressive blood cancer, characterized by restricted cellular subsets with enriched leukemia initiating cells (LICs). Recently, Ephrin receptors (Eph) were described to be highly expressed in cancer stem cells. Here, using public RNA-Seq datasets of human T-ALL, we reported that EphB6 was the only member within the Eph family overexpressed in over 260 samples. We also found the highest level of EphB6 in a minor cell subpopulation within bulk tumors of patient-derived xenografts, obtained through the injection of primary patient biopsy material into immunocompromised NOD-Scid/IL2Rγc^−/−^ (NSG) mice. Interestingly, this EphB6 positive (EphB6+) subset showed an enriched LIC activity after in vivo transplantation into NSG mice. Additionally, gene expression data at the single-cell level of primary patients’ leukemic cells revealed that EphB6 + cells were significantly selected in minimal residual disease up to 30 days from the standard treatments and characterized by high levels of markers related to cell proliferation and poor clinical outcome, such as *CCNB1* and *KIF20A*. Taken together, our data suggest that EphB6 supports LICs’ maintenance and progression in T-ALL and, thus, targeting EphB6 + cells could be therapeutically relevant for the treatment of T-ALL patients.

**Supplementary Information:**

The online version contains supplementary material available at 10.1186/s40364-023-00531-3.

## To the editor

**Fig. 1:**
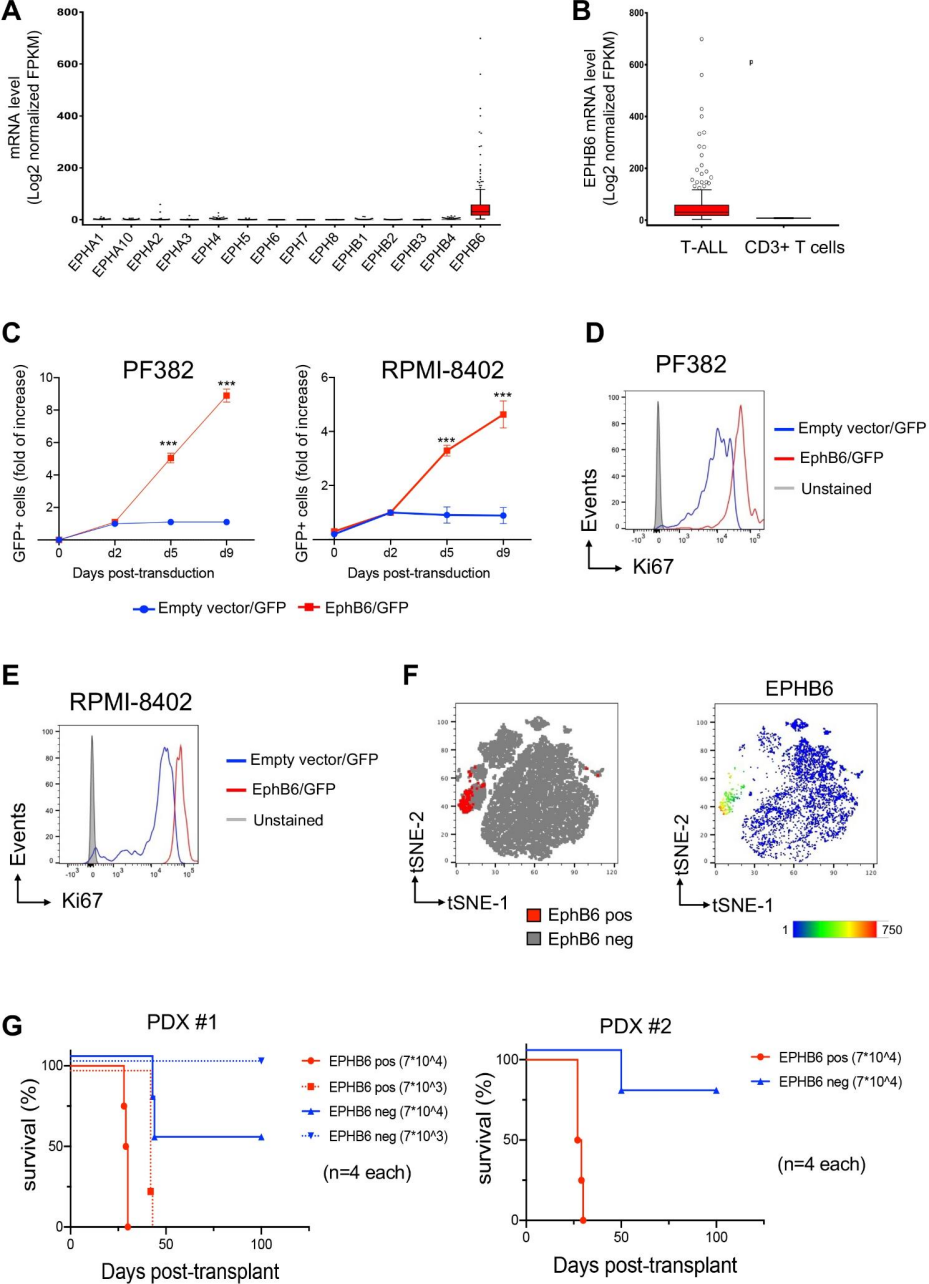
The constitutive expression of EPHB6 receptor in human T-ALL promotes in vitro proliferation and in vivo cell expansion. (**A**) Analysis of public COG TARGET dataset shows a significant enrichment of EphB6 mRNA level as compared with those of Eph receptors family in T-ALL patients (***P < 0.0001 *n* = 264, by one-way ANOVA) and (**B**) higher EphB6 expression in T-ALL cases versus CD3 + T-cells from healthy donors from the Human Protein Atlas database (***P < 0.0001 *n* = 20, by Mann-Whitney test). **(C)** Flow cytometry analysis of abundance of GFP + alive cell fraction after transduction with EPHB6 or empty (EV) lentivectors as indicated. Transduced FACS-sorted subsets of PF382 and RPMI-8402 cell lines were tracked over time at the indicated time points by flow cytometry. Alive GFP + cells were discriminated for DAPI exclusion. Means ± SD fraction of the initial transduction value are plotted for experiments performed in biological triplicate. ***, *p < 0.001 (Student’s t-test).***(D-E)** Flow cytometry analysis of Ki67 level in PF382 (D) and RPMI-8402 (E) cell lines after transduction with EPHB6 or empty (EV) lentivectors as indicated. Transduced subsets of PF382 and RPMI-8402 cell lines were valued after three days of in vitro growth. **(F)** t-SNE plots based on the immunophenotyping of M71 (PDX#1) and H3255 (PDX#2), two independent clones of PDX samples by multiparametric flow cytometry. (**G**) Survival of recipient NSG mice after transplantation with FACS-sorted EPHB6 *+* cell subsets from M71 (PDX#1) and H3255 (PDX#2) PDX samples. The cell doses injected in each of 4 recipient animals are indicated in parentheses. Two separate experiments are depicted using independent PDX clones as indicated

T-cell acute lymphoblastic leukemia (T-ALL) is a T-cell malignancy, affecting children and adults [1]. The recurrent nature of this tumor and resilience to standard chemotherapy [2], presumably due to the ineffective targeting of leukemia initiating cells (LICs) [3], concur to its lethality. Lately, it was reported that signaling mediated by EphB6 receptor interferes with the efficiency of doxorubicin treatment in T-ALL [4]. Eph receptor tyrosine kinases (Eph) family includes 16 receptors, modulated by ephrin ligands [5] and involved in tumor progression [6] and cancer stem cell maintenance [7, 8]. To address the role of Ephrin signaling in T-ALL, we evaluated the expression of different Eph receptors in 264 T-ALL patients using public available RNA-Seq datasets [9]. We found that EphB6 was the only member within the Eph group, overexpressed in the analyzed T-ALL cases (Fig. 1A) as well as in pairwise comparison with CD3 + normal T-cells (Fig. 1B) and across various human tissues and hematopoietic cells (Fig. S1).

**Fig. 2:**
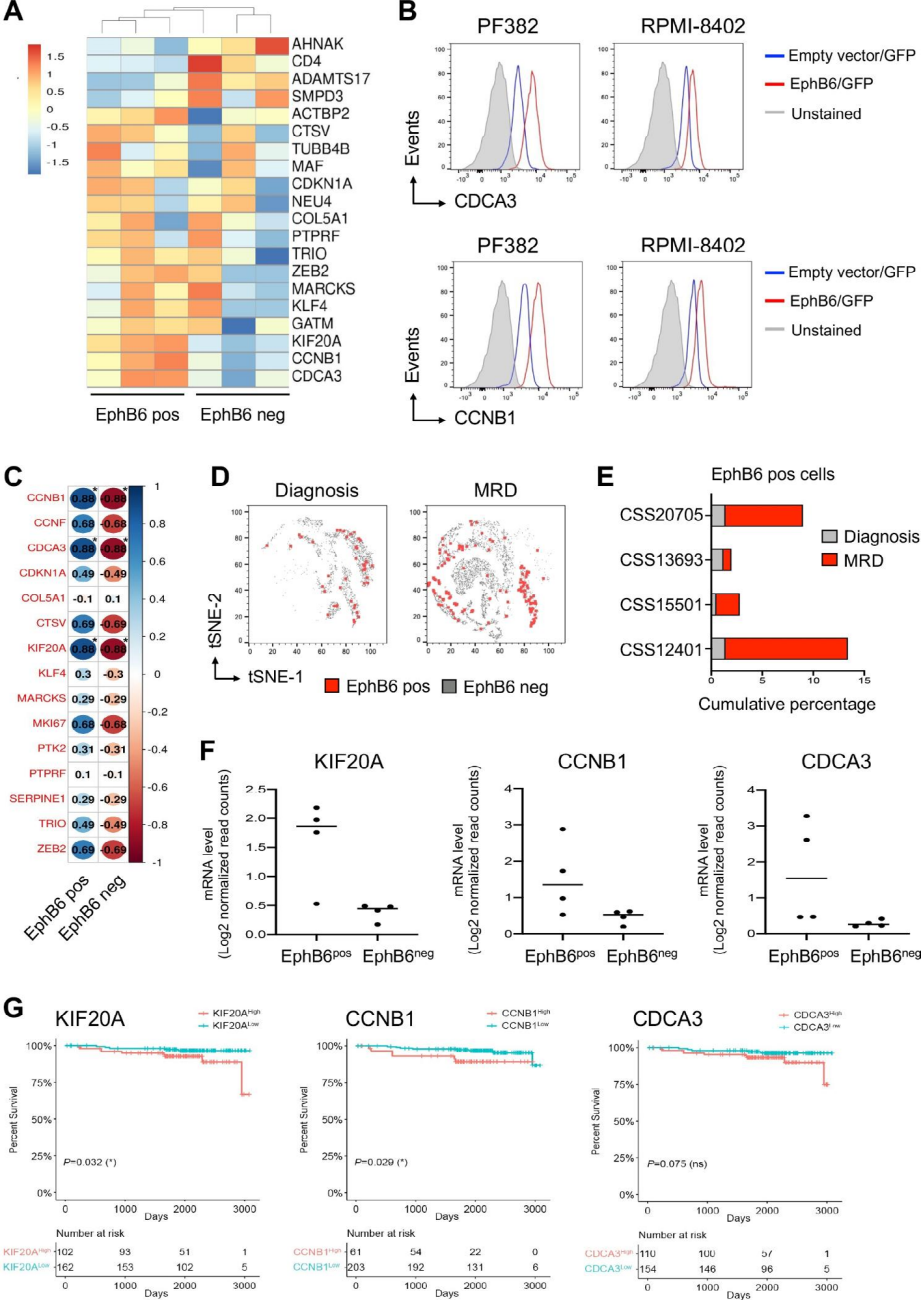
EphB6 positive cells are characterized by high transcriptional levels of genes related to cell proliferation and significantly selected in minimal residual disease (MRD) of human T-ALL. (**A**) Hierarchical clustering of EphB6 positive and EphB6 negative subpopulations using 20 differentially expressed genes from M71 (PDX#1), H3255 (PDX#2) and F1313 (PDX#3), three independent PDX clones. A dual-color code represents markers over-(red) and under-represented (blue). (**B**) Flow cytometric analysis depicting the enrichment of CCNB1 and CDCA3 in EphB6-overexpressing cells (EphB6/GFP) as compared to control (Empty vector/GFP). (**C**) Heatmap showing the Spearman’s rank correlation between the EphB6 fractions and 15 differentially expressed genes. The higher the statistical correlation the bigger the circles. A dual-color code reports positive (blue) and negative (red) correlation and color intensity represents the increase of correlation. *P < 0.05, by Student’s t-test. (**D**) tSNE plots based on the single cell RNA-Sequencing (scRNA-Seq) data from 4 primary T-ALL samples, CSS12401, CSS13693, CSS15501 and CSS20705 at the diagnosis (d0) and up to 30 days after the start of therapy (MRD) without passaging into immunocompromised mice. In the map, EphB6 negative and positive leukemia cells are colored in grey and red, respectively. (**E**) Graphical representation of cell percentage in the EphB6 positive cell population at the diagnosis (d0) and up to 30 days after the start of therapy (MRD) according to each sample group. (**F**) mRNA expression level of CDCA3, CCNB1 and KIF20A genes in EphB6 positive vs. EphB6 negative cells as determined by scRNA-seq data. ***, *P < 0.001 (Student’s t-test).* (**G**) Higher *KIF20A* and *CCNB1* genes are significantly associated with lower patients’ survival according to the COG TARGET dataset (*P* = 0.032 and *P* = 0.029 from log-rank test, *n* = 264). Kaplan-Meier survival plots shows the same trend for *CDCA3* gene although without statistical significance (*P* = 0.075, *n* = 264)

To address the potential role of EphB6 receptor in T-ALL, PF382 and RPMI-8402 cell lines were transduced with lentivectors encoding *EPHB6* gene or empty vector, as control. Interestingly, the constitutive expression of *EPHB6* primed in vitro cell expansion (Fig. 1C) and promoted cell cycling activity as determined by the high level of Ki67 nuclear protein (Fig. 1D-E) and BrdU incorporation (Fig. S2). To confirm the effectiveness of these findings in primary T-ALLs, we assessed two independent clones of patient-derived xenografts (PDX) by multiparameter flow cytometry assay including 10 fluorophore-conjugated antibodies against T-cell lineage markers (Table S1) and identified a distinct cell cluster characterized by high levels of EphB6 receptor (Fig. 1F). EphB6 positive and negative fractions of two PDXs were then FACS sorted and assayed for their in vivo leukemogenic capacity by limiting dilution transplantation assay into immunodeficient NOD-Scid/IL2Rγc^−/−^ (NSG) mice (Fig. 1G and S3). As expected, mice implanted with EphB6 + population died significantly earlier than those injected with EphB6- subset, confirming EphB6 expression as a trait of LIC. Consistently, we found that LIC frequency was higher in EphB6 + populations (Table S4).

We next conducted an RNA-seq comparative transcriptome analysis on EphB6 + and EphB6- cells of three PDXs. Genes involved in cell cycle such as *CCNB1*, and *CDCA3* were highly expressed in EphB6 + cell subsets of PDX and validated in EphB6-transduced cell lines (Fig. 2A-B and S4-5). EphB6 fractions and their transcriptomic fingerprint were also correlated to each other and a significant cogent score of correlation (r > 0.8) was found between the EphB6 + subset and the level of *KIF20A*, *CCNB1* and *CDCA3* genes (Fig. 2C). Additionally, we evaluated the transcriptional level of EphB6 transcript in single cell RNA-sequencing (scRNA-Seq) profiling generated from four different human T-ALLs at the diagnosis and minimal residual disease up to 30 days after the start of therapy without any expansion into immunocompromised NSG mice. EphB6 + cells were enriched in MRD (Fig. 2D-E) and showed high transcriptional levels of *CCNB1*, *CDCA3* and *KIF20A* genes by a meta-clustering and differential gene expression analysis (Fig. 2F and S6B)., Additionally, we found a statistically significant enrichment for the genes associated with high level of EphB6 in PDX samples and derived by the differential expression analysis of RNA-seq data sets (Fig. 2A) by gene expression enrichment analysis (GSEA) (Fig. S6A).

Finally, the predictive potential of biomarkers enriched in the high EphB6-expressing subset as compared to the depleted counterpart was assessed. We found that the high-expression levels of *KIF20A* and *CCNB1* genes were predictors of T-ALL patients’ poor prognosis with respect to their low-expressing counterpart (Fig. 2G**)** and significantly associated with the increased risk of mortality (hazard ratio: 0.70; 95% CI:0.54–0.91, *P* < 0.01 and hazard ratio: 0.53; 95% CI:0.39–0.71, *P* < 0.001, respectively). High level of *CDCA3* gene also pointed to a worse prognosis although not statistically significant (hazard ratio: 0.79; 95% CI: 0.61-1.00, *P* > 0.05) (Fig. S7-9).

Taken together, these findings suggest that EphB6 + cells might be selected after conventional treatment, altering the sensitivity of leukemia cells to different drugs [4, 10]. It was already reported that EphB6 directly modulate cellular the activation of Akt signaling in T-ALL cell lines [6, 11, 12]. In agreement with these findings, our data also suggest that EphB6 signaling alter the maintenance and progression of T-ALL cells through regulation of proliferative signaling pathways, involving genes such as *CCNB1* and *KIF20A* related to poor clinical outcome. Finally, these results suggest that LIC-enriched cell subsets may be sensitive to the EphB6 inhibitors. Nonetheless, further studies will be necessary to find the interesting possibility that therapies based on the inhibition of EphB6 signaling may improve the clinical outcomes in T-ALL patients.

### Electronic supplementary material

Below is the link to the electronic supplementary material.

Supplementary Material 1

## Data Availability

The RNA-Seq and single cell RNA-Seq (scRNA-Seq) data are accessible at NCBI SRA PRJNA972695 and PRJNA784728 respectively. For other original data, please contact the correspondent author.
