# Supplementary material for: A functional role of Ephrin type-B receptor 6 (EPHB6) in T-cell acute lymphoblastic leukemia
Source: Biomark Res. 2023 Oct 20;11:92. doi: 10.1186/s40364-023-00531-3 (PMC10588013; doi:10.1186/s40364-023-00531-3)
Supplement: Supplementary file 1 — Supplementary Material 1 [file 40364_2023_531_MOESM1_ESM.pdf]

A functional role of Ephrin type-B receptor 6 (EPHB6) in T-cell acute lymphoblastic leukemia

Mattia Colucci[1,*], Nadia Trivieri[2,*], Gandino Mencarelli[2], Elisabetta De Santis[1], Francesca Sansico[1], Francesco Tamiro[1], Alberto Visioli[3], Chiara Barile[2], Riccardo Pracella[2], Giovanni Rossi[4], Elena Binda[2,±], Vincenzo Giambra[1,*,±]

[1] Hematopathology Unit, Institute for Stem Cell Biology, Regenerative Medicine and Innovative Therapeutics (ISBReMIT), Fondazione IRCCS "Casa Sollievo della Sofferenza", Viale Padre Pio, 7, 71013, San Giovanni Rotondo (FG), Italy

[2] Cancer Stem Cells Unit, Institute for Stem Cell Biology, Regenerative Medicine and Innovative Therapeutics (ISBReMIT), Fondazione IRCCS "Casa Sollievo della Sofferenza", Viale Padre Pio, 7, 71013, San Giovanni Rotondo (FG), Italy

[3] StemGen SpA, Milan, Italy

[4] Department of Hematology and Stem Cell Transplant Unit, Fondazione IRCCS Casa Sollievo della Sofferenza, Viale Cappuccini, 1, 71013, San Giovanni Rotondo (FG), Italy

* These authors contributed equally to this work

[±]Corresponding authors

Supplemental Information including:

Supplementary Methods

Supplementary Discussion

Supplementary References

Figures S1 to S9

Tables S1 to S4

Materials and Methods

Cell culture.

Established human T-ALL cell lines, RPMI-8402 and PF382, were *in vitro* expanded in RPMI 1640 medium supplemented with 10% fetal bovine serum (FBS), 1mM sodium pyruvate, 2mM L-glutamine, and antibiotics (Invitrogen). Patient-derived xenografts were previously established by injection of primary patient biopsy material into irradiated NOD-Scid/IL2Rγc$^{-/-}$ (NSG) mice by Dr. Andrew P. Weng (BC Cancer research, Terry Fox Laboratory) as previously described (1) in accordance with the Declaration of Helsinki after approval of institutional ethics committee. Samples of PDX cells were kindly provided by Dr. Andrew P. Weng (BC Cancer research, Terry Fox Laboratory) under material transfer agreement guidelines. The M71 (PDX#1) and H3255 (PDX#2) PDX lines were both reported previously (**Table S2**) (2). Xenograft-expanded primary human T-ALLs were cultured on MS5-DL1 feeders in IMDM (GIBCO) with 10 ng/ml IL-2, 10 ng/ml IL-7 (Peprotech), and 0.75uM SR1 (StemCell Technologies) as previously described (1).

Plasmid and Viral transduction.

A cDNA encoding the human EPHB6, derived from Transomic construct (Lenti-hCMV-EPHB6-P2A-eGFP-IRES-puro, cod. #TLO2005), was expressed into a lentivector with GFP selection marker and verified by sequencing. Lentiviruses were produced by transient co-transfection of 293T cells with pCMVΔR8.74 and pCMV-VSV-G packaging/envelope vectors (3). Lentivirus was concentrated by ultracentrifugation. Viral transduction was performed by spinfection in the presence of polybrene as described (4). Virally transduced cells were sorted by flow cytometry as applicable.

Flow cytometry assays.

Human cells were stained with fluorochrome antibodies against CD3 (monoclonal antibody (SK7), Brilliant Violet 510, BD Biosciences), CD4 (monoclonal antibody (RPA-T4), Brilliant Violet 605, BD Biosciences), CD7 (monoclonal antibody (4H9), Brilliant Violet 786, BD Biosciences), TCRγ/δ (monoclonal antibody (B1), PerCP-Cy™5.5, BD Biosciences), TCRα/β (monoclonal antibody (IP26), PE, BD Biosciences), CD1a (monoclonal antibody (HI149), PE/Cyanine7, Biolegend), CD8 (monoclonal antibody (RPA-T8), APC, BD Biosciences), CD45 (monoclonal antibody (HI30), Alexa Fluor 700, BD Biosciences), CD2 (monoclonal antibody (TS1/8), APC/Fire™ 750, Biolegend), and human EPHB6 (polyclonal antibody, cod. #PA514614, ThermoFisher). An Alexa Fluor® 488 goat anti-rabbit IgG secondary antibody (cod. #A-11008, ThermoFisher) was used to detect the unconjugated antibody against EPHB6 (**Table S1**). Viable cells were distinguished by negative staining for DAPI (cod. D9542, Sigma-Aldrich), followed by debris exclusion and singlet gating based on forward and side scatter. We performed intracellular staining with an anti-KI-67 PE-Cy7-conjugated antibody (1:100 dilution; cat. #561283, BD Biosciences) as well as an anti-CDCA3 unconjugated antibody (1:50 dilution; cat. #PA5-13564, ThermoFisher) and an anti-CCNB1 unconjugated antibody (1:100 dilution; cat. #BS-0572R, ThermoFisher), followed by AlexaFluor647 goat anti-rabbit IgG secondary antibody (cod.#A-21244, ThermoFisher), after paraformaldehyde fixation and permeabilization with 90% ice-cold methanol as specified by the manufacturer. We measured cell proliferation by BrdU incorporation according to the manufacturer's instructions (BrdU kit, BD Biosciences). We performed FACS analysis and sorting on BD LSR Fortessa™ X-20, BD FACS Canto2, and MoFlo Astrios cell sorter (Beckman Coulter). Flow cytometry data were analyzed using FlowJo software (Becton Dickinson).

Human samples

The cohort of human samples involved in this study were enrolled at "Fondazione IRCCS Casa Sollievo della Sofferenza" after approval of the institutional Ethic board (Prot. N.155/CE, ICF: V1.0_16 Mag 18). All the subject agreed to participate according to the ethical guidelines of the 2013 Declaration of Helsinki with signed informed consent. Material was anonymized at the time of collection. All studies involving human material including PDX samples and cell line as well as human data, were performed in accordance with the Declaration of Helsinki after approval of the institutional Ethic board.

In vivo transplantation assays.

NOD-Scid/IL2Rγc$^{-/-}$ (NSG) mice were housed in specific pathogen-free facilities at the Plaisant s.r.l. (Rome, Italy). The animal experiments were performed after protocol approval by the Institutional Review Boards of the Italian Ministry of Health, according to the Italian Regulation for Animal Health and Welfare (n 509/2018-PR). Immunodeficient (NOD.Cg-Prkdcscid Il2rgtm1Wjl/SzJ, or NSG; RRID: IMSR_JAX:005557) xenograft recipient mice were 7-17 weeks of age. Male and female animals were represented in balanced proportion when in-house colony stock availability necessitated using mixed sex recipients.

Gene expression and sequencing data analysis.

Total RNA was isolated by TRIzol™ (Invitrogen) following manufacturer's standard protocol (TRIzol™ Reagent; Invitrogen) from FACS-sorted EPHB6+ and EPHB6- cell subsets from 3 independent clones of PDX samples. Library preparation and sequencing of ribodepleted total

RNA was performed in service at the BC Cancer Genome Sciences Centre (Vancouver), reaching about 25 million reads for sample. The quality of RNA was assessed by QuibitRNA assay and Agilent Bioanalyzer. Trim galore, a wrapper around Cutadapt version 3.7 and FastQC, was used for the adapter trimming and to do further quality assessment of raw file (5). The splice-aware genome aligner STAR was used to align adapter-trimmed paired-end reads to the human reference genome (hg19). The alignment post-processing was then conducted using the Picard tool (https://broadinstitute.github.io/picard/) with the "Picard markDuplicates" command to mark duplicate reads. Differential expression analysis between EphB6 positive and EphB6 negative samples was carried out by LPEseq, which is able to perform well on limited number of samples, in particular non-replicated samples (6).

To identify the disease and function, we performed the predictions analysis with Ingenuity Pathway Analysis (IPA; Qiagen, http://www.ingenuity.com/) and R software(7, 8), using significantly differentially expressed genes (P-value<0.05, $\log_2$(fold-change)>|1.3|). The biological functions with activating or inhibiting z-score threshold ≥ 2 or ≤ -2 and adjusted P-value (*q-value*) <0.05 was considered significant. Diseases and functions were graphically represented with "ggh4x" R package (v.4.1.2). The RNA-Seq data are accessible at NCBI SRA PRJNA972695.

Analysis of public datasets.

Eph receptors mRNA levels in T-ALL patients (n=264), healthy controls (CD3+ normal T-cells) (n=20) and human tissues were downloaded from the Therapeutically Applicable Research to Generate Effective Treatments (TARGET) phase3 T-ALL database (https://target-data.nci.nih.gov/Public/ALL/SAMPLE_MATRIX/Phase3/) (COG TARGET dataset(9)) and

from. Human Protein Atlas dataset (https://www.proteinatlas.org/) respectively. EphB6 expression levels in human hematopoietic cells at different maturation stages were downloaded from the Leukemia MILE study (www.bloodspot.eu)(10). Data were graphed in box plots and Jitter strip chart as log_2-normalized Fragments per Kilobase Million (FPKM) counts, log_2-normalized Transcripts Per Million (TPM) and log_2 expression values. The "survminer" R package was used to create COG TARGET dataset's Kaplan-Maier survival curves of patients and to calculate the best cut-off to identify high or low mRNA expression groups as in (11).

Statistical analysis.

Statistical tests were performed by R and GraphPad Prism v.7.0 software according to the variance and distribution of data. Spearman correlation coefficient was used to evaluate correlation using "corrplot" and "Hmisc" R packages. *P*-value<0.05 was considered statistically significant. A multivariate Cox proportional hazard regression model analysis for the selected genes was employed on multiple predictor variables such as EphB6 level, minimal residual disease (MRD) at day 30 and ages, gender. Data relative to MRD and age were grouped in three intermediate clusters and analyzed with the "survival" R package. *P*-value<0.05 was considered significant. Heatmaps were produced with “pheatmap” R package. Survival plots were estimated by Kaplan-Meier method and survival distribution compared by Log-rank test. A probability value <0.05 was considered significant.

Multivariate Cox proportional hazard model applied on multiple predictor variables (*EphB6* Status, MRD_Day_29 and Ages, divided in three intermediate groups, gender and selected Genes Status).

Quantitative real-time RT-PCR

Total RNA was extracted using the Direct-zol RNA Miniprep kits (Zymo Research) with a genomic DNA removal step and cDNA was obtained using SuperScriptIII^{TM} Reverse Transcriptase (ThermoFisher Scientific) and 1 μg of total RNA that was primed with oligo-dT for cDNA synthesis. qPCR reactions were run in triplicate using ITaq^{TM} Universal SYBR Green Supermix (Bio-Rad), recorded in real-time (Quantstudio5 System Real-Time PCR, Thermo Fisher Scientific), and normalized to *GAPDH* as endogenous control. The gene expression profile was delineated by the $2^{-\Delta Ct}$ method of relative control and mRNA levels.

Single cell RNA-sequencing (scRNA-Seq)

Leukemia cells from cryopreserved bone marrow samples of T-ALL patients (**Table S3**) were isolated as CD45+CD3-CD99+CD7+ cell fraction by FACS-sorting as previously reported (12). Isolated cells from each sample were labeled with the BD Single-Cell Multiplexing Kit (cat. 633781, BD Biosciences) for 1 hour following the manufacturer's protocol. The cell viability and concentration were determined with the BD Rhapsody Scanner system after staining with viability dyes, Calcein AM (1:200 dilution; cat. #C1430, ThermoFisher) and DRAQ7^{TM} (1:200 dilution; cat. #564904, BD Biosciences), and incubation for 5 min at 37°C. Cells were counted using the Improved Neubauer Hemocytometer (INCYTO). Afterward leukemia cells for each sample at d0 and d30 were pooled equally in 650ml cold BD Sample Buffer and three BD Rhapsody cartridges were loaded with 10,000 pooled cells for single cell separation. Single cells were isolated using Single-Cell Capture and cDNA Synthesis with the BD Rhapsody Express Single-Cell Analysis System according to the manufacturer's recommendations (BD Biosciences). Based on the number of viable cells revealed and captured on the beads, the final resuspension volume was calculated

to subsample and sequence about 4,000 cells. Whole transcriptome and Sample Tag amplification was performed with the BD Rhapsody Whole Transcriptome Amplification Kit (cat. #633774) following the manufacturer's instructions. Unwanted PCR products and other small molecules were excluded performing a side cleanup using the AMPure XP Beckman magnetic beads (cat. #A63880, Beckman Coulter). DNA quantity and quality control were performed using the Qubit™ dsDNA HS Assay Kit (cat. # Q32851, ThermoFisher Scientific) and the electrophoresis system Agilent 2200 TapeStation, cartridge (cat. #5067-5584). Sequencing was performed in paired-end mode (2*75 cycles) on NextSeq 500 System (Illumina) with the NextSeq 500/550 High Output Kit v2.5 (150 Cycles) chemistry to reach a depth of 75,000 reads for WTA and 500 reads for SMK per cell for a total of 80,000 reads per cell on average. Sequencing data were processed on the Seven Bridges Platform (https://www.sevenbridges.com/) for sample demultiplexing and generation of expression sparse matrixes. Briefly, a quality sequencing step was performed to filter out reads with a Phred quality score below 20, reads with a single type of nucleotide and short reads (less than 60 bases for R1 and less then 42 for R2). UMI counting was done including sequencing reads correction, recursive substation error correction (RSEC) and distribution-based error correction (DBEC). Accepted reads were aligned to the reference genome (GRCh38) for identification and quantification and subsequently the count genes matrix was generated. Highly dimensional Sc-RNA-Seq data were analyzed using the SeqGeq software (Becton Dickinson) for visualization, clustering and differential expression analysis. Only cells with mitochondrial read rate ≤ 30%, detectable genes ≥ 200 and genes expressed in 10 or more cells were considered passing the QC and further analyzed using functions provided with the Seurat library. In the end, we counted 6,322 total cells. Data were log normalized regressing out both the number of counts and percentage of reads aligning to mitochondrial genes. Clusters were allocated to cell

populations based on gene markers using uncentered correlation and centroid linkage. The scRNA-Seq data are accessible at NCBI SRA PRJNA784728.

Discussion

T-cell acute lymphoblastic leukemia (ALL) is an aggressive malignancy, characterized by an uncontrolled expansion of immature T-cells (13-16). Refractory and relapsed cases are present in both pediatric and adult T-ALL patients (15-17) with a 5-year overall survival (OS) that significantly decreases with the age (18). Therefore, novel prognostic biomarkers and more effective and safe treatments are needed for T-ALL patients at high risk. In the last years, subsets with asymmetrically enriched leukemia initiating cell (LIC) activity have been described in both human and mouse models of T-ALL by our group and others (4, 19-23), emphasizing the functional relevance of regulatory pathways in the maintenance and expansion of LIC-enriched subsets (1, 24-27).

In this study, we explored the role of Eph signaling in the progression of human T-ALLs. We report that the EphB6 gene is highly and specifically expressed in human T-ALL in line with previous findings (28). Interestingly, we also found that the EphB6+ cell subsets of human T-ALLs were also highly enriched in LICs and that the constitutive expression of EPHB6 receptor in T-ALL cell line promoted cell growth, highlighting the relevance of Eph signaling in T-ALL progression. This not only supported the key role reported for this receptor in T cell developmental

processes (29), but also suggested that EphB6 could be a crucial therapeutic target in the development of personalized clinical strategies.

Furthermore, through a scRNA-Seq profiling of tumor cells, derived from T-ALL patients at the diagnosis and minimal residual disease (MRD) at 30 days after the start of the therapy and without any expansion into immunocompromised mice, we outline that *EPHB6* gene is mostly enriched in MRD. Notably, these EphB6 positive subpopulations were characterized by a distinctive transcriptional signature related to cell cycle progression, microtubule dynamics, cell proliferation, tumorigenesis and migration, supporting the idea that EphB6 signaling enforces proliferative and infiltrative potential in primary human T-ALL cells.

Taken together, these findings suggest that EphB6 positive cells might be selected after conventional treatment by protective local tumor microenvironment, altering the sensitivity of leukemia cells to different drugs (28, 30). It was reported that signaling mediated by EPHB6 receptor increases the sensitivity of T-ALL cells to DNA damage induced by doxorubicin in cell culture and in xenograft animals (28). Inversely, our data show that EPHB6 expression in RPMI-8402 cell line promotes *in vitro* resistance to dexamethasone (Fig. S9), which is an agonist of the glucocorticoid receptor (GR) (31), suggesting that EphB6 positive cells might differently respond to distinct drug treatments. Interestingly, EphB6 directly modulate cellular the activation of Erk kinases in lung cancer cells (32, 33) as well as Akt signaling in T-ALL cell lines (34-36). Activation of PI3K-Akt was indeed previously described to promote growth of established T-cell leukemias (37-39) and leukemia-initiating cell (LIC) activity in T-ALL (40). In agreement with these findings, our data also suggest that EphB6 signaling alter the maintenance and progression

of tumor cells in T-ALL through regulation of proliferative signaling pathways, involving genes related to poor clinical outcome, such as *CCNB1*, *CDCA3* and *KIF20A*.

Finally, these results highlight the potential clinical relevance of inhibition of EphB6 signaling in T-ALL suggesting that LIC-enriched cell subsets may be sensitive to the EphB6 inhibitors by blocking the LIC activity of minimal residual disease and directly reducing the rates of disease relapse in T-ALL. This approach might be more effective in the cure of high risk and more chemo-resistant T-ALL cases who are currently overtreated, by reducing adverse side effects. Nonetheless, further studies will be necessary to find the interesting possibility that therapies based on the inhibition of EphB6 signaling and/or activity of EphB6 positive cells may improve the clinical outcomes in T-ALL patients.

Supplementary References

1. Giambra V, Gusscott S, Gracias D, Song R, Lam SH, Panelli P, et al. Epigenetic Restoration of Fetal-like IGF1 Signaling Inhibits Leukemia Stem Cell Activity. Cell Stem Cell. 2018.

2. Yost AJ, Shevchuk OO, Gooch R, Gusscott S, You MJ, Ince TA, et al. Defined, serum-free conditions for in vitro culture of primary human T-ALL blasts. Leukemia. 2013;27(6):1437-40.

3. Dull T, Zufferey R, Kelly M, Mandel RJ, Nguyen M, Trono D, Naldini L. A third-generation lentivirus vector with a conditional packaging system. J Virol. 1998;72(11):8463-71.

4. Giambra V, Jenkins CR, Wang H, Lam SH, Shevchuk OO, Nemirovsky O, et al. NOTCH1 promotes T cell leukemia-initiating activity by RUNX-mediated regulation of PKC-theta and reactive oxygen species. Nat Med. 2012;18(11):1693-8.

5. M. M. Cutadapt Removes Adapter Sequences From High-Throughput Sequencing Reads. EMBnetjournal. 2011;17(1):10-2.

6. Gim J, Won S, Park T. LPEseq: Local-Pooled-Error Test for RNA Sequencing Experiments with a Small Number of Replicates. PLoS One. 2016;11(8):e0159182.

7. Binda E, Visioli A, Giani F, Lamorte G, Copetti M, Pitter KL, et al. The EphA2 receptor drives self-renewal and tumorigenicity in stem-like tumor-propagating cells from human glioblastomas. Cancer Cell. 2012;22(6):765-80.

8. Visioli A, Giani F, Trivieri N, Pracella R, Miccinilli E, Cariglia MG, et al. Stemness underpinning all steps of human colorectal cancer defines the core of effective therapeutic strategies. EBioMedicine. 2019;44:346-60.

9. Liu Y, Easton J, Shao Y, Maciaszek J, Wang Z, Wilkinson MR, et al. The genomic landscape of pediatric and young adult T-lineage acute lymphoblastic leukemia. Nat Genet. 2017;49(8):1211-8.

10. Bagger FO, Sasivarevic D, Sohi SH, Laursen LG, Pundhir S, Sonderby CK, et al. BloodSpot: a database of gene expression profiles and transcriptional programs for healthy and malignant haematopoiesis. Nucleic acids research. 2016;44(D1):D917-24.

11. Trivieri N, Visioli A, Mencarelli G, Cariglia MG, Marongiu L, Pracella R, et al. Growth factor independence underpins a paroxysmal, aggressive Wnt5a(High)/EphA2(Low) phenotype in glioblastoma stem cells, conducive to experimental combinatorial therapy. J Exp Clin Cancer Res. 2022;41(1):139.

12. Dworzak MN, Froschl G, Printz D, Zen LD, Gaipa G, Ratei R, et al. CD99 expression in T-lineage ALL: implications for flow cytometric detection of minimal residual disease. Leukemia. 2004;18(4):703-8.

13. Pui CH, Robison LL, Look AT. Acute lymphoblastic leukaemia. Lancet. 2008;371(9617):1030-43.

14. Uckun FM, Sensel MG, Sun L, Steinherz PG, Trigg ME, Heerema NA, et al. Biology and treatment of childhood T-lineage acute lymphoblastic leukemia. Blood. 1998;91(3):735-46.

15. Pui CH, Evans WE. Treatment of acute lymphoblastic leukemia. N Engl J Med. 2006;354(2):166-78.

16. Pui CH, Pei D, Campana D, Bowman WP, Sandlund JT, Kaste SC, et al. Improved prognosis for older adolescents with acute lymphoblastic leukemia. J Clin Oncol. 2011;29(4):386-91.

17. Marks DI, Rowntree C. Management of adults with T-cell lymphoblastic leukemia. Blood. 2017;129(9):1134-42.

18. Guru Murthy GS, Pondaiah SK, Abedin S, Atallah E. Incidence and survival of T-cell acute lymphoblastic leukemia in the United States. Leuk Lymphoma. 2019;60(5):1171-8.

19. Chiang MY, Shestova O, Xu L, Aster JC, Pear WS. Divergent effects of supraphysiologic Notch signals on leukemia stem cells and hematopoietic stem cells. Blood. 2013;121(6):905-17.

20. Guo W, Lasky JL, Chang CJ, Mosessian S, Lewis X, Xiao Y, et al. Multi-genetic events collaboratively contribute to Pten-null leukaemia stem-cell formation. Nature. 2008;453(7194):529-33.

21. Tatarek J, Cullion K, Ashworth T, Gerstein R, Aster JC, Kelliher MA. Notch1 inhibition targets the leukemia-initiating cells in a Tal1/Lmo2 mouse model of T-ALL. Blood. 2011;118(6):1579-90.

22. Cox CV, Martin HM, Kearns PR, Virgo P, Evely RS, Blair A. Characterization of a progenitor cell population in childhood T-cell acute lymphoblastic leukemia. Blood. 2007;109(2):674-82.

23. Chiu PP, Jiang H, Dick JE. Leukemia-initiating cells in human T-lymphoblastic leukemia exhibit glucocorticoid resistance. Blood. 2010;116(24):5268-79.

24. Armstrong F, Brunet de la Grange P, Gerby B, Rouyez MC, Calvo J, Fontenay M, et al. NOTCH is a key regulator of human T-cell acute leukemia initiating cell activity. Blood. 2009;113(8):1730-40.

25. Tremblay M, Tremblay CS, Herblot S, Aplan PD, Hebert J, Perreault C, Hoang T. Modeling T-cell acute lymphoblastic leukemia induced by the SCL and LMO1 oncogenes. Genes Dev. 2010;24(11):1093-105.

26. Giambra V, Jenkins CE, Lam SH, Hoofd C, Belmonte M, Wang X, et al. Leukemia stem cells in T-ALL require active Hif1alpha and Wnt signaling. Blood. 2015;125(25):3917-27.

27. Panelli P, De Santis E, Colucci M, Tamiro F, Sansico F, Miroballo M, et al. Noncanonical beta-catenin interactions promote leukemia-initiating activity in early T-cell acute lymphoblastic leukemia. Blood. 2022.

28. El Zawily A, McEwen E, Toosi B, Vizeacoumar FS, Freywald T, Vizeacoumar FJ, Freywald A. The EphB6 receptor is overexpressed in pediatric T cell acute lymphoblastic leukemia and increases its sensitivity to doxorubicin treatment. Sci Rep. 2017;7(1):14767.

29. Shimoyama M, Matsuoka H, Tamekane A, Ito M, Iwata N, Inoue R, et al. T-cell-specific expression of kinase-defective Eph-family receptor protein, EphB6 in normal as well as transformed hematopoietic cells. Growth Factors. 2000;18(1):63-78.

30. Toosi BM, El Zawily A, Truitt L, Shannon M, Allonby O, Babu M, et al. EPHB6 augments both development and drug sensitivity of triple-negative breast cancer tumours. Oncogene. 2018;37(30):4073-93.

31. Madamsetty VS, Mohammadinejad R, Uzieliene I, Nabavi N, Dehshahri A, Garcia-Couce J, et al. Dexamethasone: Insights into Pharmacological Aspects, Therapeutic Mechanisms, and Delivery Systems. ACS Biomater Sci Eng. 2022;8(5):1763-90.

32. Yu J, Bulk E, Ji P, Hascher A, Koschmieder S, Berdel WE, Muller-Tidow C. The kinase defective EPHB6 receptor tyrosine kinase activates MAP kinase signaling in lung adenocarcinoma. Int J Oncol. 2009;35(1):175-9.

33. Fan YH, Ding HW, Kim D, Liu JY, Hong JY, Xu YN, et al. The PI3Kalpha inhibitor DFX24 suppresses tumor growth and metastasis in non-small cell lung cancer via ERK inhibition and EPHB6 reactivation. Pharmacol Res. 2020;160:105147.

34. Maddigan A, Truitt L, Arsenault R, Freywald T, Allonby O, Dean J, et al. EphB receptors trigger Akt activation and suppress Fas receptor-induced apoptosis in malignant T lymphocytes. J Immunol. 2011;187(11):5983-94.

35. Luo H, Wan X, Wu Y, Wu J. Cross-linking of EphB6 resulting in signal transduction and apoptosis in Jurkat cells. J Immunol. 2001;167(3):1362-70.

36. Strozen TG, Sharpe JC, Harris ED, Uppalapati M, Toosi BM. The EphB6 Receptor: Kinase-Dead but Very Much Alive. Int J Mol Sci. 2021;22(15).

37. Chiarini F, Fala F, Tazzari PL, Ricci F, Astolfi A, Pession A, et al. Dual inhibition of class IA phosphatidylinositol 3-kinase and mammalian target of rapamycin as a new therapeutic option for T-cell acute lymphoblastic leukemia. Cancer Res. 2009;69(8):3520-8.

38. Sanda T, Li X, Gutierrez A, Ahn Y, Neuberg DS, O'Neil J, et al. Interconnecting molecular pathways in the pathogenesis and drug sensitivity of T-cell acute lymphoblastic leukemia. Blood. 2010;115(9):1735-45.

39. Cullion K, Draheim KM, Hermance N, Tammam J, Sharma VM, Ware C, et al. Targeting the Notch1 and mTOR pathways in a mouse T-ALL model. Blood. 2009;113(24):6172-81.

40. Medyouf H, Gusscott S, Wang H, Tseng JC, Wai C, Nemirovsky O, et al. High-level IGF1R expression is required for leukemia-initiating cell activity in T-ALL and is supported by Notch signaling. J Exp Med. 2011;208(9):1809-22.

41. Coustan-Smith E, Mullighan CG, Onciu M, Behm FG, Raimondi SC, Pei D, et al. Early T-cell precursor leukaemia: a subtype of very high-risk acute lymphoblastic leukaemia. The Lancet Oncology. 2009;10(2):147-56.

42. Basso G, Veltroni M, Valsecchi MG, Dworzak MN, Ratei R, Silvestri D, et al. Risk of relapse of childhood acute lymphoblastic leukemia is predicted by flow cytometric measurement of residual disease on day 15 bone marrow. J Clin Oncol. 2009;27(31):5168-74.

43. Sulis ML, Williams O, Palomero T, Tosello V, Pallikuppam S, Real PJ, et al. NOTCH1 extracellular juxtamembrane expansion mutations in T-ALL. Blood. 2008;112(3):733-40.

44. Kuzilkova D, Bugarin C, Rejlova K, Schulz AR, Mei HE, Paganin M, et al. Either IL-7 activation of JAK-STAT or BEZ inhibition of PI3K-AKT-mTOR pathways dominates the single-cell phosphosignature of ex vivo treated pediatric T-cell acute lymphoblastic leukemia cells. Haematologica. 2022;107(6):1293-310.

45. Kucukcankurt F, Erbilgin Y, Firtina S, Hatirnaz Ng O, Karakas Z, Celkan T, et al. PTEN and AKT1 Variations in Childhood T-Cell Acute Lymphoblastic Leukemia. Turk J Haematol. 2020;37(2):98-103.

46. Diccianni MB, Yu J, Hsiao M, Mukherjee S, Shao LE, Yu AL. Clinical significance of p53 mutations in relapsed T-cell acute lymphoblastic leukemia. Blood. 1994;84(9):3105-12.

47. Asnafi V, Buzyn A, Le Noir S, Baleydier F, Simon A, Beldjord K, et al. NOTCH1/FBXW7 mutation identifies a large subgroup with favorable outcome in adult T-cell acute lymphoblastic leukemia (T-ALL): a Group for Research on Adult Acute Lymphoblastic Leukemia (GRAALL) study. Blood. 2009;113(17):3918-24.

48. Panelli P, De Santis E, Colucci M, Tamiro F, Sansico F, Miroballo M, et al. Noncanonical beta-catenin interactions promote leukemia-initiating activity in early T-cell acute lymphoblastic leukemia. Blood. 2023;141(13):1597-609.

49. Hu Y, Smyth GK. ELDA: extreme limiting dilution analysis for comparing depleted and enriched populations in stem cell and other assays. Journal of immunological methods. 2009;347(1-2):70-8.

Supplementary Figures

Fig. S1.

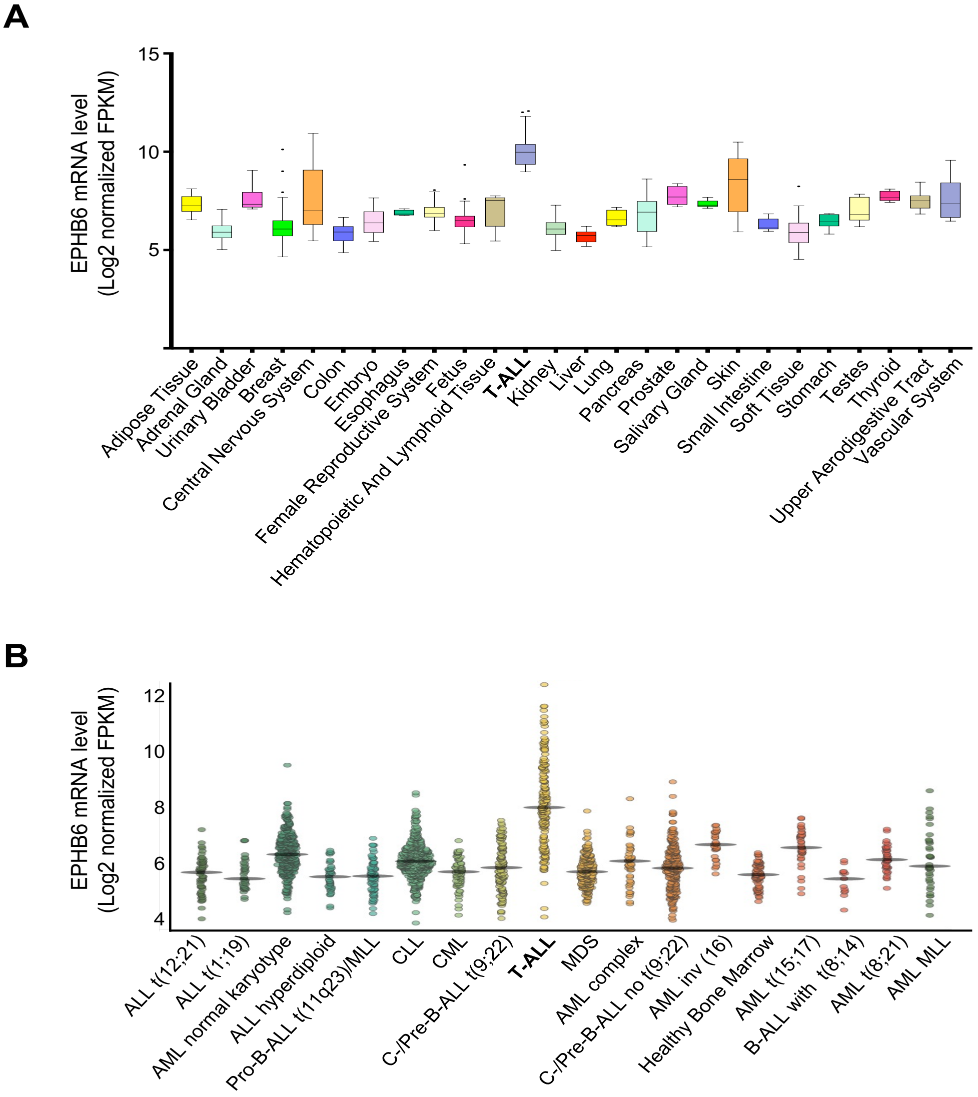

Figure S1. The *EPHB6* gene is highly and specifically expressed in human T-ALL.

(**A**) Within the public Human Protein Atlas dataset and (**B**) the Leukemia MILE study EphB6 expression is significantly higher in T-ALL tissues (****P*<0.0001, *n*=15, by Kruskal-Wallis test) and hematopoietic cells (****P*<0.0001, *n*=174, by Kruskal-Wallis test).

Fig. S2.

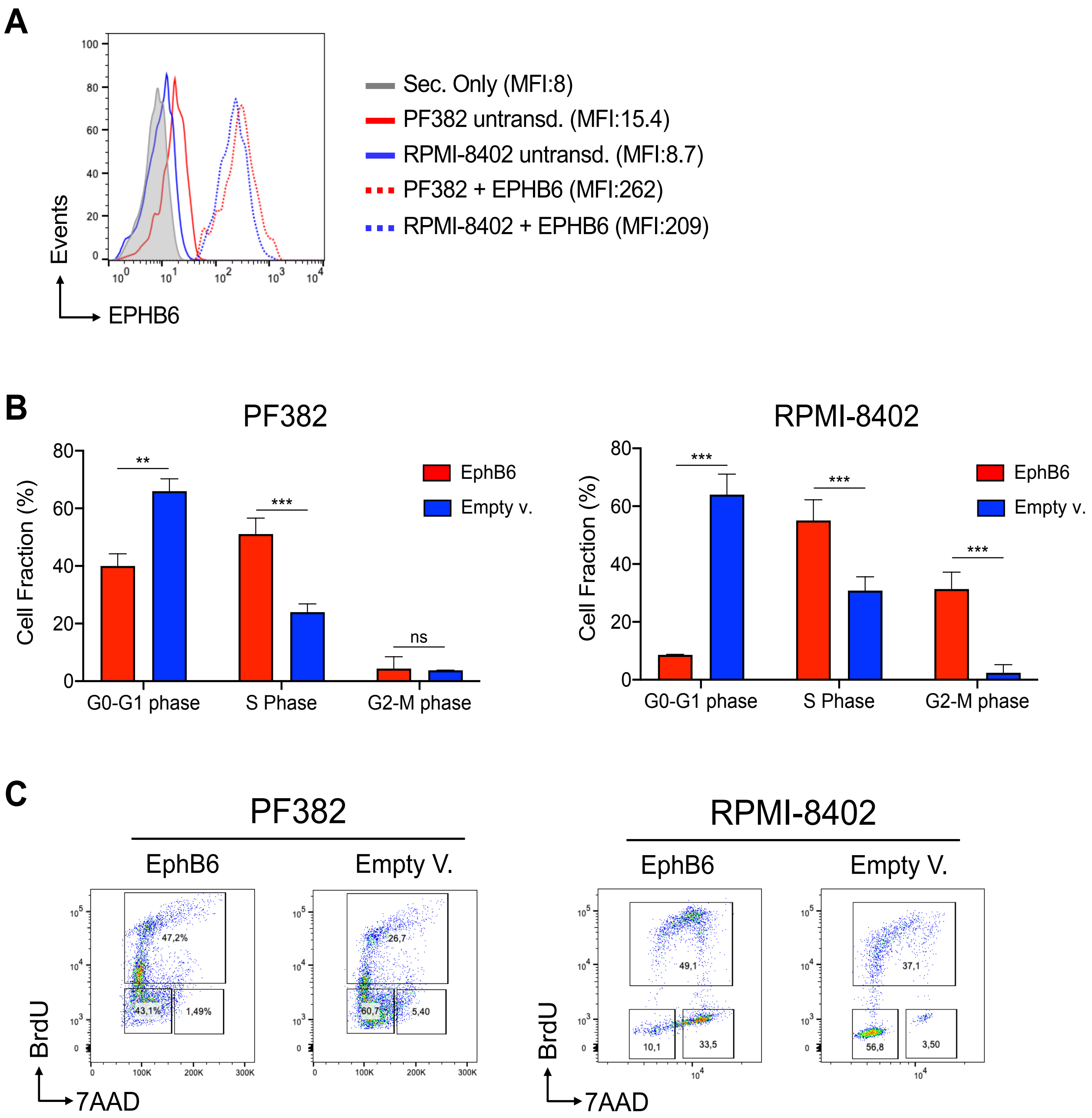
A
Events
EPHB6
Sec. Only (MFI:8)
PF382 untransd. (MFI:15.4)
RPMI-8402 untransd. (MFI:8.7)
PF382 + EPHB6 (MFI:262)
RPMI-8402 + EPHB6 (MFI:209)
B
PF382
RPMI-8402
Cell Fraction (%)
EphB6
Empty v.
G0-G1 phase
S Phase
G2-M phase
**
***
ns
C
PF382
RPMI-8402
EphB6
Empty V.
BrdU
7AAD
47,2%
43,1%
1,49%
26,7
60,7
5,40
49,1
10,1
33,5
37,1
56,8
3,50

Figure S2. The constitutive expression of EPHB6 gene promotes cell proliferation in T-ALL cell lines.

(A) Flow cytometric analysis of protein level of EPHB6 in PF382 and RPMI-8402 T-ALL cell lines, transduced by lentiviruses encoding EPHB6 gene or untransduced as control. Transduced GFP+ alive cells were stained with an anti-EPHB6 primary antibody and evaluated after seven days from the transduction for DAPI exclusion by flow cytometry. The level of mean fluorescence intensity (MFI) of EPHB6 signal is indicated in brackets. **(B)** Cell cycle analysis by BrdU incorporation in human PF382 and RPMI-8402 cell lines, following transduction with EPHB6 or empty lentivectors as indicated. Transduced cells were measured after three days of *in vitro* growth by flow cytometry. The graphs report the result of two independent experiments performed in triplicate. (**B**) Flow cytometry plots of BrdU assay used to generate the bar charts reported above. *ns, not significant; **, p<0.05; **, p<0.01; ***, p<0.001 (Student's t-test).*

Fig. S3.

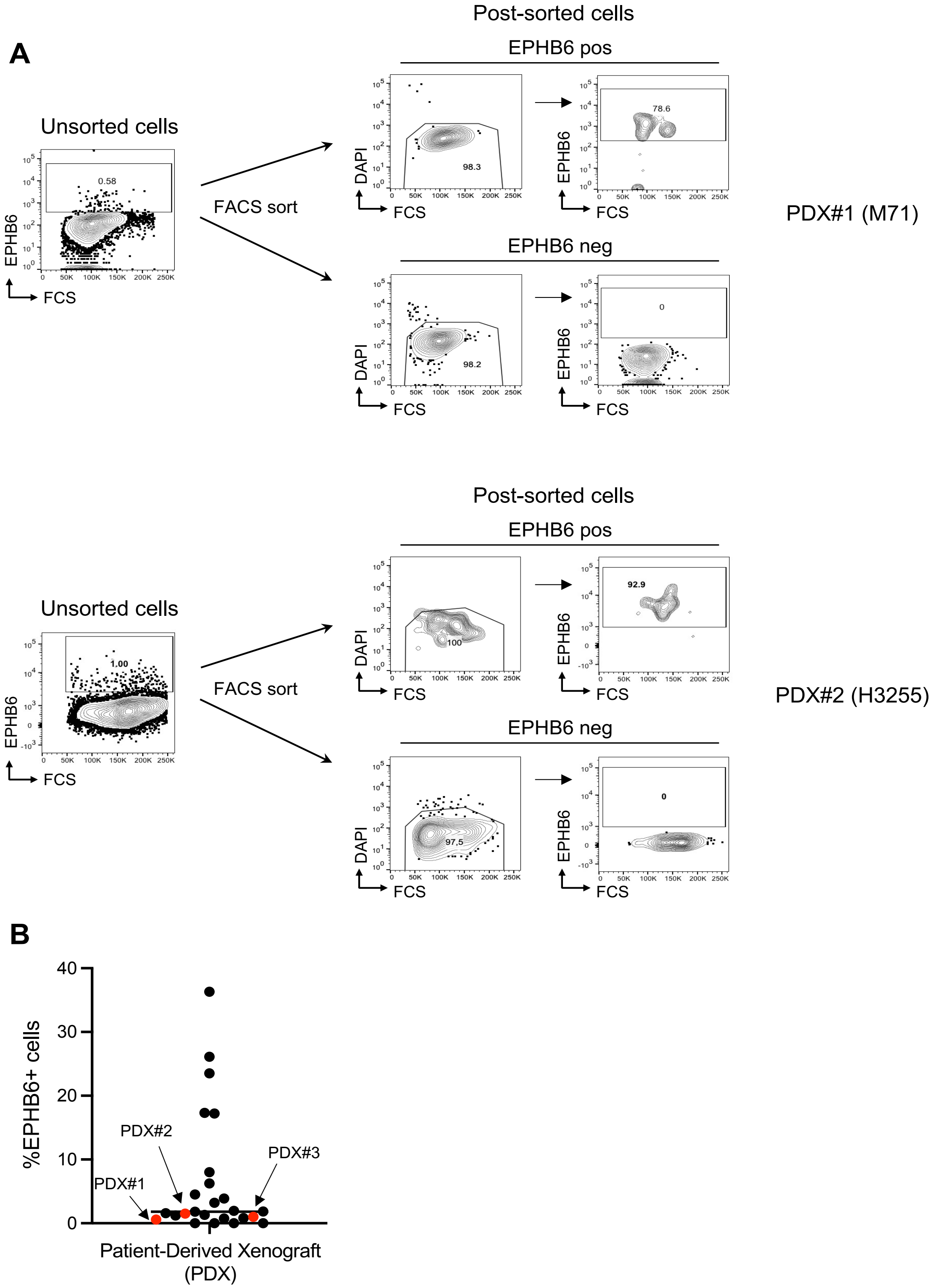
A
Post-sorted cells
EPHB6 pos
EPHB6 neg
Unsorted cells
FACS sort
EPHB6
DAPI
FCS
0.58
98.3
78.6
98.2
0
PDX#1 (M71)
1.00
100
92.9
97.5
0
PDX#2 (H3255)
B
%EPHB6+ cells
PDX#1
PDX#2
PDX#3
Patient-Derived Xenograft (PDX)

Figure S3. Related to Figure 1G,

(A) Flow cytometry plots showing the EPHB6+ cell fraction before and after FACS-sorting of leukemia cells of M71 (PDX#1) and H3255 (PDX#2) patient-derived xenografts (PDXs) before the transplant into recipient mice at limiting dilution.

(B) Distribution of EPHB6+ cell subsets in 25 independent PDX clones, as determined by flow cytometry. The PDX samples assessed in this study are highlighted in red.

Fig. S4.

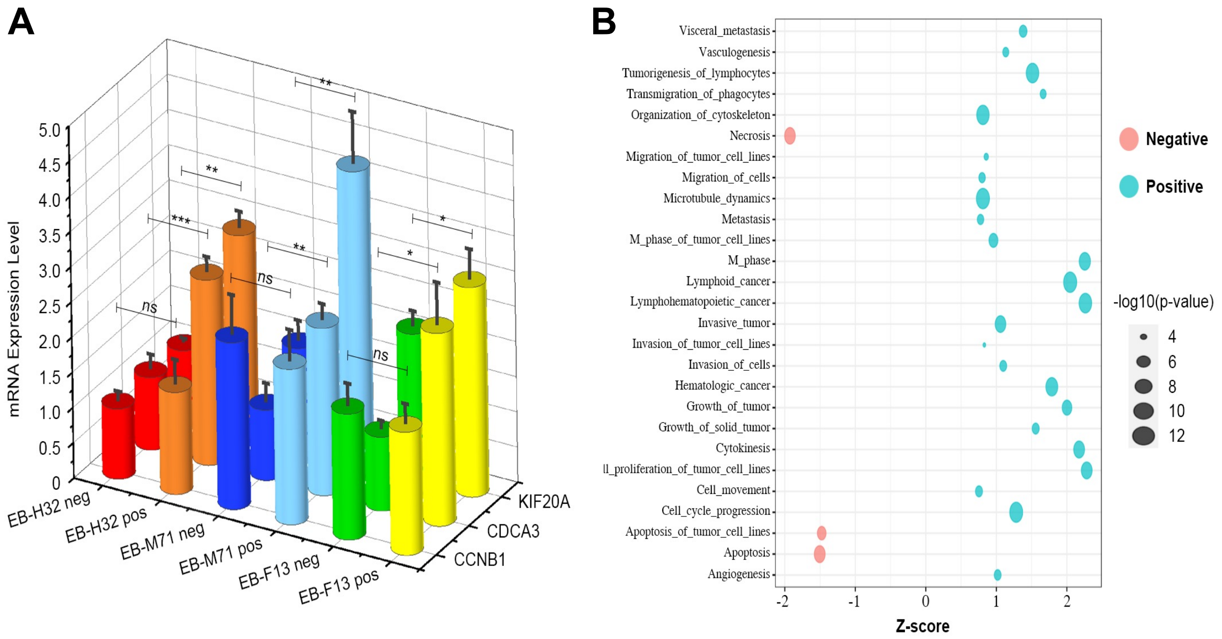

Figure S4. EphB6 positive cells are characterized by high transcriptional levels of genes related to cell proliferation.

(A) Quantification of mRNA expression level of EphB6-signature genes by qPCR analysis on EphB6 positive and EphB6 negative fractions from various cell lines. **P<0.001, *P<0.01, *P<0.05, by Welch's t-test. (**B**) When compared to their EphB6 negative siblings, EphB6 positive subset overexpressed genes related to cell proliferation, cell cycle, cell movement and invasion, as depicted by bubble plots. Colors blue and red indicate positive and negative z-score, respectively. The positive z-scores mean functional activation. The higher the statistical significance the bigger the circles.

Fig. S5.

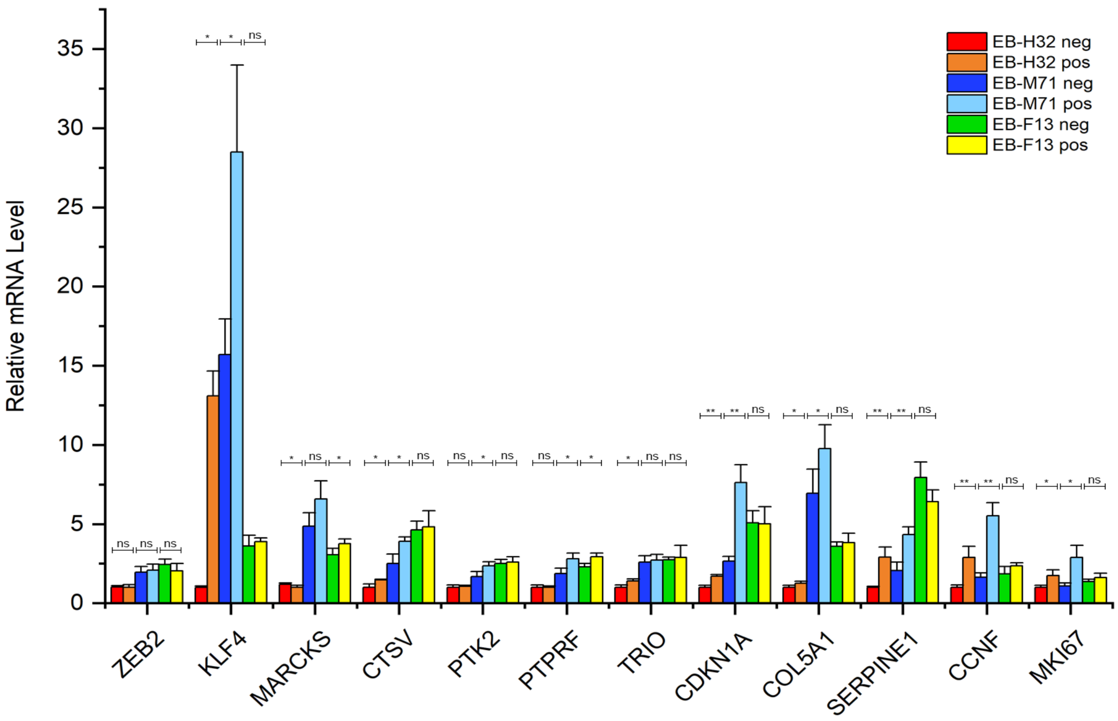

Figure S5. EphB6 overexpression drives a distinctive transcriptional fingerprint.

Quantification of mRNA expression level of EphB6-signature genes by qPCR analysis on EphB6 positive and EphB6 negative fractions from various cell lines. **P<0.001, *P<0.01, *P<0.05, by Welch's t-test.

Fig. S6.

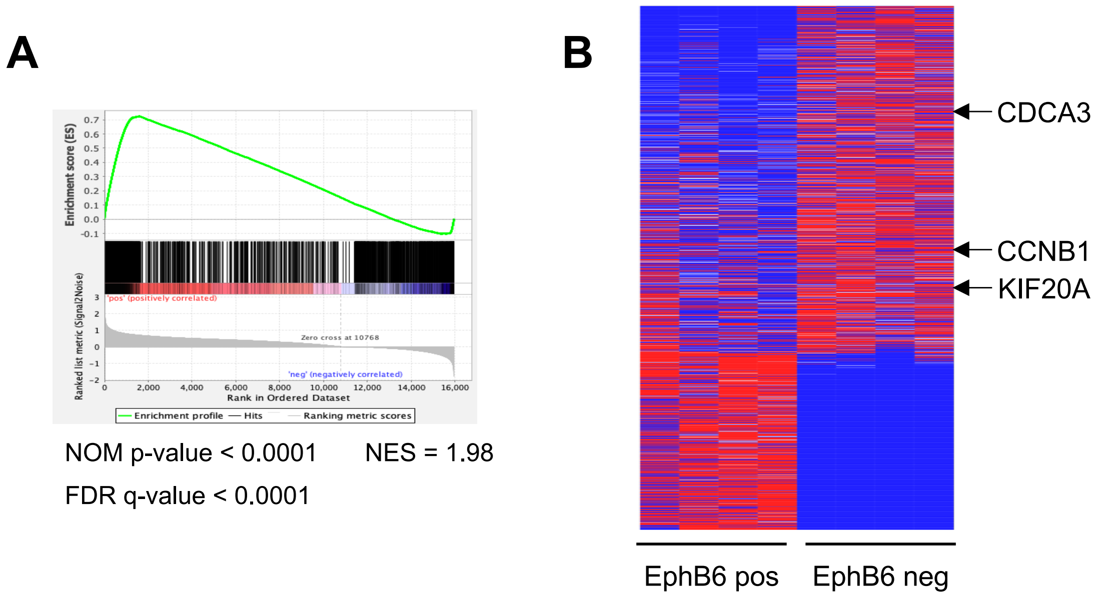

Figure S6. EphB6 positive cells are significantly selected in minimal residual disease (MRD) of human T-ALL.

(A) Gene set enrichment analysis (GSEA). In the panel, the gene signature was derived by the differential expression analysis of RNA-seq data sets from Figure 2A (n=1672 genes). All genes were ranked for differential expression in EphB6 positive vs. EphB6 negative cells of primary T-ALL sample from the scRNA-seq profiling. NES, normalized enrichment score; NOM, nominal; FDR, false discovery rate. **(B)** Expression heat map of gene expression scRNA-Seq data in each of the EphB6 positive and negative cell subsets, hierarchically clustered into the 4 primary T-ALL samples after the start of therapy (MRD). Differentially expressed genes, scaled with mean = 0 and SD = 1, are represented (adjusted p-value ≤ 0.1 and LogFC ≤ -0.5) by Morpheus-Broad Institute software (https://software.broadinstitute.org/morpheus).

Fig. S7.

A

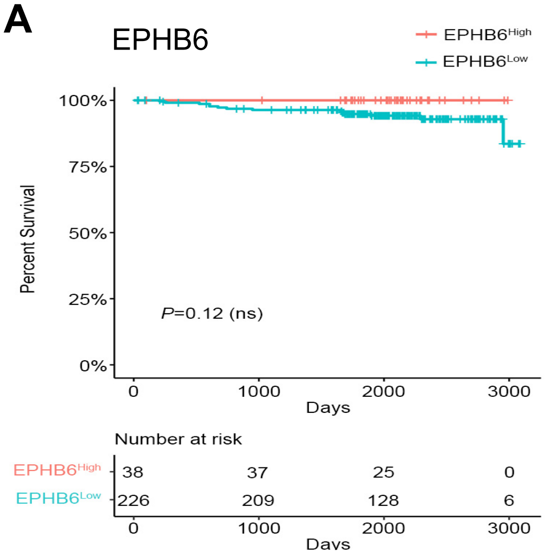
EPHB6
EPHB6High
EPHB6Low
100%
75%
50%
25%
0%
Percent Survival
P=0.12 (ns)
0
1000
2000
3000
Days
Number at risk
EPHB6High 38 37 25 0
EPHB6Low 226 209 128 6
Days

B

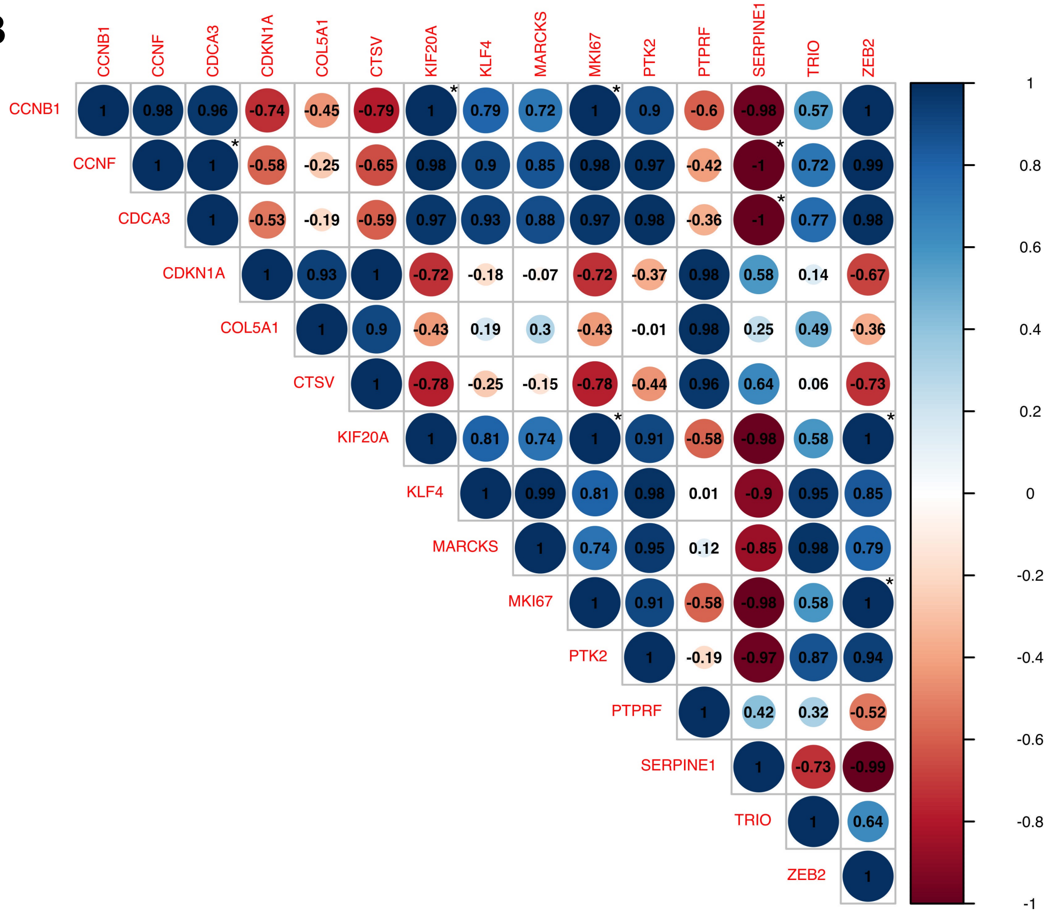
CCNB1
CCNF
CDCA3
CDKN1A
COL5A1
CTSV
KIF20A
KLF4
MARCKS
MKI67
PTK2
PTPRF
SERPINE1
TRIO
ZEB2
1
0.8
0.6
0.4
0.2
0
-0.2
-0.4
-0.6
-0.8
-1

Figure S7. Kaplan-Meier curve and gene expression correlation across Mullighan dataset. (**A**) Kaplan Meier plots showing T-ALL patients' overall survival in COG TARGET dataset (*n*=264) associated with high level of *EphB6* expression level (*n*=38) and low level of *EphB6* (*n*=226) (Log-rank test, *P*=0.12). (**B**) Heatmap representing the Pearson coefficient between each pair of genes in *EphB6* positive samples. A strong positive or negative correlation is shown by values near +1 and blue circles or near -1 and red circles, respectively. Larger or smaller circles and color intensity show higher or lower absolute correlations. *$P<0.05$, Student's t-test.

Fig. S8.

KIF20A

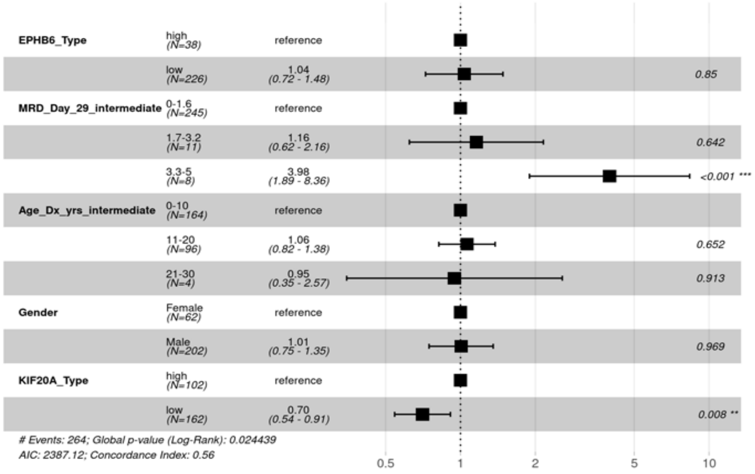

CCNB1

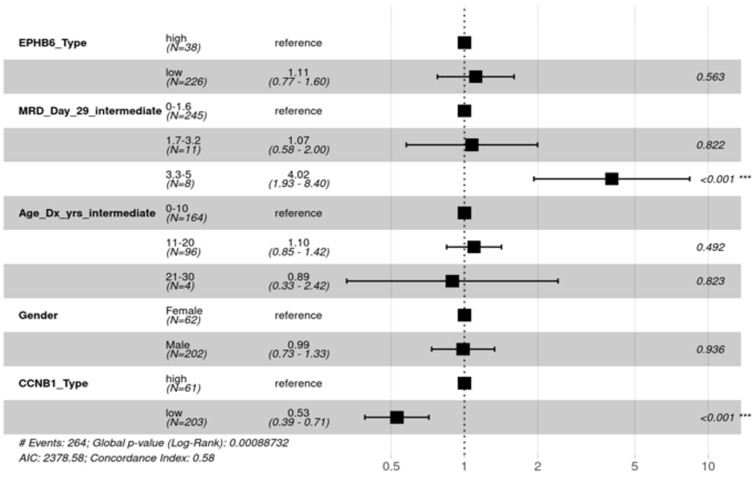

CDCA3

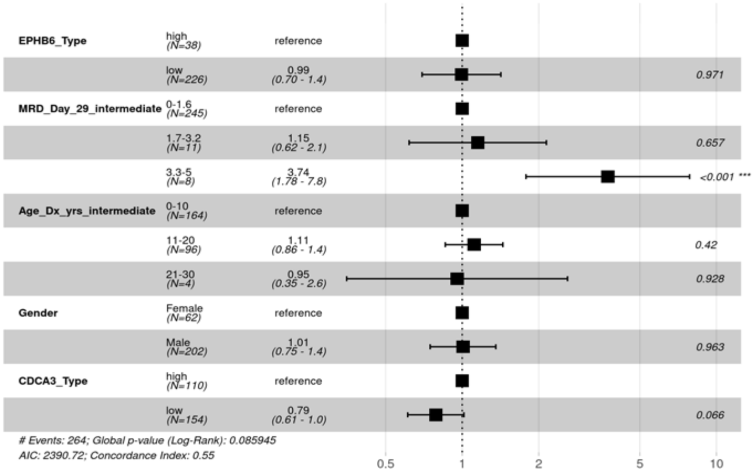

Figure S8. Potential prognostic value of *KIF20A* and *CCNB1* genes.

Multivariate Cox regression analysis showing that low level of *KIF20A* and *CCNB1* genes is significantly associated with a reduced risk of mortality (hazard ratio < 1, $P<0.01$ and $P<0.001$, respectively). Multivariate Cox regression analysis indicates a hazard ratio<1 also for low levels of *CDCA3* gene although without statistical significance.

Fig. S9.

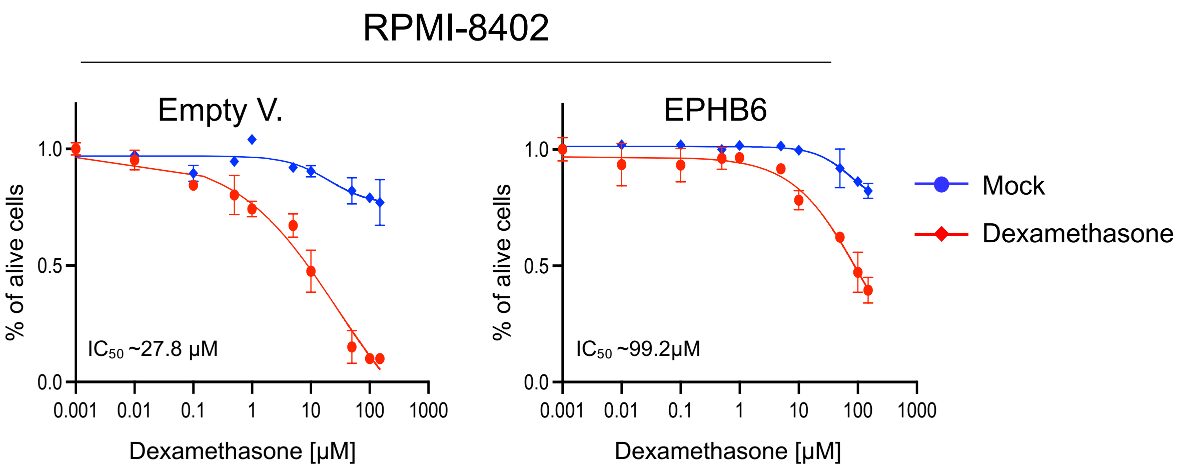

Figure S9. Representative lethal dose-response curve for Dexamethasone in EPHB6-transduced RPMI-8402 cell lines.

Cytotoxicity assays were performed on RPMI-8402 cell lines, overexpressing EPHB6 gene function or empty vector (EV) as control. Viable cells were measured after 72h of treatment with DMSO (mock) or Dexamethasone at the indicated concentrations by flow cytometry analysis for DAPI exclusion. The graphs report the result of two independent experiments performed in duplicates (mean ± SD are plotted).

Supplementary Table

Table S1.

Laser	Detector	Fluorochrome	Marker
405	450/50	DAPI	Viability
	525/50	BV510	CD3
	610/20	BV605	CD4
	780/60	BV786	CD7
488	450/50	DAPI	Viability
	530/30	AF488	EPHB6 + anti-Rabbit
	710/50	PerCP-Cy5.5	TCRγ/δ
561	585/15	PE	TCRα/β
	780/60	PeCy7	Cd1a
640	670/14	APC	CD8
	730/45	AF700	CD45
	780/60	APC/fire	CD2

Table S1. Panel of cell surface markers and fluorophore-conjugated antibodies used in the multiparameter flow cytometry assessment of patient-derived xenografts as reported in Figure 1F. APC, allophycocyanine; AF, Alexa Fluor; BV, Brilliant™ Violet; PE, phycoerythrin.

Table S2.

	PDX#1	PDX#2	PDX#3
Sample ID	M71	H3255	F1313
Diagnosis/Relapse	Diagnosis	Diagnosis	Relapse
Age	<18	26	<18
Gender	Unknown	Male	Unknown
NOTCH1 HD mutation	L1586P	WT	L1586P
NOTCH1 PEST mutation	WT	A2480V	WT
PTEN mutation	WT	WT	WT
FBXW7 mutation	WT	R479Q	WT
Tested In vivo (Fig. 1G)	Yes	Yes	No
RNA-Seq (Fig. 2A)	Yes	Yes	Yes

Table S2. Demographic, clinical and genetic features of considered patient-derived xenografts (PDXs).

The characteristics of patient-derived xenografts (PDXs) were previously reported in Yost A. et al.(2) HD, Heterodimerization domain; WT, wild-type.

Table S3.

	CSS12401	CSS15501	CSS13693	CSS20705
Gender	M	M	M	F
Age at diagnosis (years)	17	30	36	55
NOTCH1 (exons 26-27-34)	nd	wt	TAD mut	TAD mut
FBXW7 (exons 9-12)	nd	wt	mut	wt
PTEN (exon 7)	nd	wt	wt	wt
P53 (exons 4-5)	nd	wt	wt	wt
IL7R (exon 6)	nd	mut	wt	wt
Year of diagnosis	2019	2019	2020	2020
WBC/mm^3 at diagnosis		3,800	65,000	64,000
EGIL score	5	5	5	5
ETP immunophenotype	no	no	no	yes
MRD at day 30	<0.01%	1.78%	2.64%	4.17%
Therapy	AIEOP -BFM AL	prot GIMEMA LAL1913	prot GIMEMA LAL1913	prot GIMEMA LAL1913
vital status	alive	alive	alive	alive
Year of last follow-up	2023	2023	2023	2023
Karyotype			46,XY	46,XX,del(12)(q24::qter)[9]

Table S3. Demographic, clinical and genetic features of considered T-ALL patients in the scRNA-Seq assay.

Early T-cell (ETP) phenotype was assessed according to Coustan-Smith E. et al.(41) The percentage of minimal residual disease (MRD) in the bone marrow at day 30 was determined by flow cytometry according to Basso G. et al.(42) Mutations on the exons of indicated genes were determined as previously reported (43-48). TAD, Transactivation domain; HD, Heterodimerization domain; wt, wild-type; nd, not determined.

Table S4.

Donor (clone)	Donor Type	Recipient ID (Strain)	Injected Cells after Sorting (% purity)	Injected Cell Dose	Clinical Outcome	Latency (days)	% CD45+ Spleen Cells at Necropsy	% EPHB6+ Leukemic Spleen Cells at Necropsy
M71-10	PDX#1	1 (NSG)	$CD45^+$**EPHB6+** (78.6)	70,000	Leukemia	28	81	1.1
M71-10	PDX#1	2 (NSG)	$CD45^+$**EPHB6+** (78.6)	70,000	Leukemia	29	78	2.3
M71-10	PDX#1	3 (NSG)	$CD45^+$**EPHB6+** (78.6)	70,000	Leukemia	30	82	5.2
M71-10	PDX#1	4 (NSG)	$CD45^+$**EPHB6+** (78.6)	70,000	Leukemia	30	nd	nd
M71-10	PDX#1	1 (NSG)	$CD45^+$**EPHB6+** (78.6)	7,000	Leukemia	42	78	3.2
M71-10	PDX#1	2 (NSG)	$CD45^+$**EPHB6+** (78.6)	7,000	Leukemia	42	82	3.9
M71-10	PDX#1	3 (NSG)	$CD45^+$**EPHB6+** (78.6)	7,000	Leukemia	42	nd	nd
M71-10	PDX#1	4 (NSG)	$CD45^+$**EPHB6+** (78.6)	7,000	Leukemia	43	88	2.1
M71-10	PDX#1	1 (NSG)	$CD45^+$**EPHB6-** (100)	70,000	Leukemia	43	78	0.5
M71-10	PDX#1	2 (NSG)	$CD45^+$**EPHB6-** (100)	70,000	Leukemia	44	81	0.2
M71-10	PDX#1	3 (NSG)	$CD45^+$**EPHB6-** (100)	70,000	NED (d100)			
M71-10	PDX#1	4 (NSG)	$CD45^+$**EPHB6-** (100)	70,000	NED (d100)			
M71-10	PDX#1	1 (NSG)	$CD45^+$**EPHB6-** (100)	7,000	NED (d100)			
M71-10	PDX#1	2 (NSG)	$CD45^+$**EPHB6-** (100)	7,000	NED (d100)			
M71-10	PDX#1	3 (NSG)	$CD45^+$**EPHB6-** (100)	7,000	NED (d100)			
M71-10	PDX#1	4 (NSG)	$CD45^+$**EPHB6-** (100)	7,000	NED (d100)			
H3255-1	PDX#2	1 (NSG)	$CD45^+$**EPHB6+** (92.9)	70,000	Leukemia	27	nd	nd
H3255-1	PDX#2	2 (NSG)	$CD45^+$**EPHB6+** (92.9)	70,000	Leukemia	27	88	4.1
H3255-1	PDX#2	3 (NSG)	$CD45^+$**EPHB6+** (92.9)	70,000	Leukemia	29	72	3.2
H3255-1	PDX#2	4 (NSG)	$CD45^+$**EPHB6+** (92.9)	70,000	Leukemia	30	75	5.1
H3255-1	PDX#2	1 (NSG)	$CD45^+$**EPHB6-** (100)	70,000	Leukemia	50	nd	nd
H3255-1	PDX#2	2 (NSG)	$CD45^+$**EPHB6-** (100)	70,000	NED (d100)			
H3255-1	PDX#2	3 (NSG)	$CD45^+$**EPHB6-** (100)	70,000	NED (d100)			
H3255-1	PDX#2	4 (NSG)	$CD45^+$**EPHB6-** (100)	70,000	NED (d100)			

Table S4, related to Figure 1G. Summary of transplant experiments with M71-10 (PDX#1) and H3255-1 (PDX#2), two independent clones of PDX samples.

% CD45+ cell fraction is among gated viable spleen cells; % EPHB6+ is among gated viable human CD45+ cells. Specifically, the calculated LIC frequency in EPHB6 positive cells of was lower than 10,907 (95% CI: 1 in 1-10,907) and lower than 109,322 (95% CI: 1 in 1-109,322) in PDX#1 and PDX#2 respectively and in EPHB6 negative cells was 1 in 115,486 (95% CI: 1 in 28870-461,970) and 1 in 100,989 (95% CI: 1 in 24,562-415,226) in PDX#1 and PDX#2 respectively, as evaluated by ELDA analysis at the following link (https://bioinf.wehi.edu.au/software/elda/) as previously described (49). Animals with a CD45+ cell fraction less than 0.01% in bone marrow, spleen and/or peripheral blood were considered leukemia-free as determined by flow cytometry. NED (dX), no evidence of disease as of X days post-transplant; ND, not determined.
